# Diarrhea incidence and intestinal infections among rotavirus vaccinated infants from a poor area in Brazil: a spatial analysis

**DOI:** 10.1186/1471-2458-14-399

**Published:** 2014-04-24

**Authors:** Claudimary Bispo Santos, Karina Conceição GM Araújo, Anne Jardim-Botelho, Márcio Bezerra Santos, Alda Rodrigues, Silvio Santana Dolabella, Ricardo Queiroz Gurgel

**Affiliations:** 1 Núcleo de Pós-Graduação em Medicina, Universidade Federal de Sergipe, Aracaju, SE, Brazil; 2 Programa de Pós-Graduação em Biologia Parasitária, Universidade Federal de Sergipe, Aracaju, SE, Brazil

**Keywords:** Spatial analysis, Acute diarrhea, Environmental conditions, Intestinal infections, Rotavirus

## Abstract

**Background:**

Acute diarrhea is the second leading cause of mortality among children under 5 years of age in developing countries. The pathogen most strongly associated with diarrhea is rotavirus followed by enteric pathogens such as bacteria, helminthes and protozoan. Adequate sanitation and water supply contribute to decrease acute diarrhea incidence of most etiologic agents, although vaccination remains the most important intervention to control rotavirus acute diarrhea. This study aimed to describe environmental conditions and analyze spatially the acute diarrhea and intestinal infection among rotavirus vaccinated infants from Laranjeiras-Sergipe, Brazil.

**Methods:**

Children were enrolled between 2 and 11 months of age and followed through 12 months. Demographic, socioeconomic and environmental data were obtained from a questionnaire, and immunization data were obtained from children vaccination card. Children stool samples were collected each month in order to run laboratory analyses. The household spatial localization was obtained by using a Global Positioning System (GPS). Spatial analysis was performed using the TerraView computer program and Kernel intensity estimation.

**Results:**

A total of 1,113 stool samples were collected with 80 being diarrhea associated. Diarrhea incidence rate was 0.5 ± 1.0 episodes/child/year. The overall infection rates by *Ascaris lumbricoides*, *Endolimax nana*, *Giardia lamblia* and rotavirus were 5.1%, 3.0%, 0.9% and 2.6%, respectively. 3.8% of diarrhea-associated stool samples were positive for rotavirus and 11.3% were positive for helminths and protozoans. There were some changes on spatial distribution of intestinal infections and diarrhea episodes along the four trimesters evaluated.

**Conclusions:**

The studied infants live equally in precarious conditions of sanitation which probably explain the significant rates of parasitic infections appearing in early life. The low acute diarrhea incidence in the studied rotavirus vaccinated population and the low number of symptomatic rotavirus infection may indicate vaccination efficacy to prevent acute diarrhea among young children in a poor environmental sanitary setting.

## Background

Brazil now lives a transitional socioeconomic condition, oscillating from “emergent leader” [1] to a BRICS’s (Brazil, Russia, India, China, South Africa) group with a possible “lost track” situation [2]. In reality, there are remaining important social problems that directly impact public health [3]. These problems contribute to a continued high incidence of infectious and parasitic diseases, especially in populations with limited access to adequate sanitation, revealing the degree of environmental contamination by potential human pathogens [4].

Parasitic intestinal infections are considered important because of the frequency with which they may produce damage, including some delay in physical and intellectual development, particularly in younger age groups [5,6].

The spatial distribution of intestinal parasites varies within and between spatial clusters. Households within large clusters have higher prevalence of intestinal parasitic infections than smaller clusters due to larger possibility of contamination when with sewage deficit, contaminated soil, poor water quality, malnutrition, limited host resistance, poorer socio-economic status and poor hygiene conditions [7,3].

Infectious and parasitic diseases transmission is associated to the environment, making its understanding very important. It may be important to know where and how frequently such infections occur, to plan strategies of control [8].

Viral agents are important and widely distributed (including developed and developing regions) causal agents of diarrhea in different age groups. Among them, the rotavirus represents the most common cause of childhood diarrhea in industrialized and developing countries and contributes significantly to infant mortality in the last ones [9]. The vaccination is the main control strategy [10], since environmental interventions such as sanitation and good water quality substantially prevent bacterial and parasitic infections, but are ineffective against rotavirus [11].

Brazil has the largest cohort of rotavirus vaccinated individuals worldwide and introduced very early after approval (March 2006) the Rotarix® (GSK Biologicals, Rixensart, Belgium) vaccine. Studies have shown progressive reduction in the proportion of rotavirus in diarrhea cases in Sergipe, since the introduction of rotavirus vaccine (24% in 2006, 9.5% in 2007 and 7.4% in 2008) [12] and in Brazil have impacted with the reduction of diarrhea mortality and hospital admissions [13].

After vaccine introduction, diarrhea epidemiology have definitely changed, but there were very few studies, if any, evaluating other causes occurrence and environmental involvement and contribution to the disease distribution and profile. This study aimed to describe environmental conditions and analyze spatially the acute diarrhea and intestinal infection among rotavirus vaccinated infants from Laranjeiras, Sergipe, Brazil.

## Methods

This is a prospective survey describing the association of spatial and environmental conditions and the incidence of acute diarrhea and intestinal infections of young children in Laranjeiras, Sergipe, Northeast, Brazil. Laranjeiras is a 27,000 inhabitant’s municipality with 7,404 (100%) families assisted by the Family Health Program (PSF), a regional hospital and 13 health units [14]. The town is situated in a low income agricultural area producing sugar cane for the processing of sugar and alcohol. All children born at term (≥37 weeks) between April 2009 and January 2010 were eligible for inclusion. Children were enrolled between the ages of 2 and 11 months. After obtaining the list of all birth registrations in Laranjeiras’ town hall, babies were located by contacting the town’s health promoters of the *Programa Saúde da Família* (Family Health Programme, PSF). PSF promoters keep registers of all residents under their responsibility and are part of the National Health Service. Birth registration is compulsory in the country, resulting in near complete birth registration and hospital delivery is also nearly universal. Sewage facilities are considered inadequate for the majority of the population, with 20% of the families (1,402 families) having open sewage discard to the street and 5,480 (74%) families using cesspit and 522 (6%) with no protective system. Water supply is better distributed, with 72% of the families having access to piped treated water, while 24% use water from wells and 4% from other natural sources [14].

We assessed the population with children aging 2 to 11 months of life living in the municipality with the collaboration of the Health Department of Laranjeiras, which released the list of all live births in the period of study (n = 222). The eligible children were identified using the central municipality records and all parents/guardians were invited to attend information meetings at each Health Unit corresponding to the area where the family was registered. After written concordance they were interviewed using a questionnaire to obtain demographic, socioeconomic and environmental information of their households. Children’s vaccination cards were checked to confirm the dates when the 1st and 2nd doses of rotavirus vaccine had been administered.

Monthly stool samples were requested as well as samples during any acute diarrhea episode according to World Health Organization (WHO) criteria [15]. A project employee visited households every two weeks to reinforce with mothers (and collect samples if available) to provide monthly stool samples. Besides monthly samples, they were asked to take to the health center they were registered, stool sample each time the child had diarrhea. The health center staff was asked to immediately contact the project member who collected the sample and take it to a fridge located at the central health department building. The stool samples were taken to Aracaju twice a week, aliquoted and frozen (-70°C) for later study of rotavirus at the Central Laboratory of Sergipe (LACEN). Parasitic analyzes were performed within 6 hours of arrival at the laboratory of the Federal University of Sergipe (FUS). Helminth and protozoan infections were assessed by sedimentation or method of Hoffmann technique [16] and Rotavirus infection by ELISA (Rotaclone, Meridian Diagnostics, Cincinnati, OH). Stool cultures was performed for all diarrhea episodes and for the first 586 routine samples using Cary-Blair transport media sowed in MacConkey agar, *Salmonella-Shigella* (SS) agar and enriched in tetrathionate broth.

The spatial data collection was performed by the absolute method with an instant positioning of a point, using the Global Positioning System (GPS), GARMIN model, for the spatial location of households and of each Health Unit corresponding to the area [17]. Geoprocessing Information System was used in the generation of maps with the cartographic base of the Town of Laranjeiras (Digital Urban Charter of the Town of Laranjeiras IBGE - COD280360), available in digital media. Based on the descriptive attributes related to the positivity of enteric infections (rotavirus, helminthes and protozoa) and diarrhea episodes of each child represented by their housings, it was estimated a model of spatial and temporal dependence that allowed the interpolation of the surface [18,19] shown in the maps corresponding to the quarters: 1st quarter (March to May 2010); 2nd quarter (June to August/2010); 3rd quarter (September to November/2010), 4th quarter (December to February/2011) and throughout the entire study period (March 2010 to February/2011).

The study was approved by the Ethics Committee of the Federal University of Sergipe. Children were enrolled after written informed consent from parents or guardians was obtained.

### Analysis

The spatial data were imported into the software TerraView 4.1 (http://www.inpe.br/) and analyzed by the exploratory interpolation technique, Kernel estimative. From the statistical smoothing or mitigation this technique generated a surface of density for visual detection of hot areas or “hot spots”, defined as a concentration of events that indicate somehow the clustering in a spatial distribution. The distribution of points was transformed into a continuous area of risk for the occurrence of enteric infections in the studied location. This procedure allowed filtering the variability of a set of data, without, however, changing the essential form of their local characteristics [19].

Relative and absolute values of frequency were used to describe the studied population. The rate of intestinal infection was obtained from calculations of the cumulative incidence at every 3 months of follow-up. Multiple logistic regression analysis was used to determine the variables that most influenced the episodes of diarrhea and the enteric infections, whose measure was calculated from the logistic model odds ratio (OR) with confidence interval (CI) of 95%. The significance level of *p* ≤ 0.05 was applied. The data analyzes were performed in the BioStat Program version 5.0 and SPSS version 17.0.

## Results

Out of the 222 children born in Laranjeiras during the period, 173 (77.9%) children could be accessed and were enrolled at the first meetings. Of these 130 (75%) remained during the full 12 months of follow-up. Among the 43 children who left the study, 29 (16.8%) moved to another city and 13 (7.5%) dropped out. During the study, one patient died not associated to diarrhea or enteric infection.

Most participants lived in inadequate sanitary conditions, whereas 76% lived in areas lacking sanitary sewage, using cesspits, and 50% of mothers stated there were open ditches with wastewater near their residences. The majority of families (73%) had piped water inside their homes, 65% arising from the public water company; and 73% lived in homes made of bricks (Table 1).

**Table 1:** Environmental conditions of the children’s housings in the study, Laranjeiras, Sergipe, Brazil, 2010-11

**ENVIRONMENTAL CONDITIONS**	**n = 173**	**%**
**Types of housings**		
Brick	126	73
Mud	7	4
Block	40	23
**Piped water**		
Yes, inside the home	126	73
Yes, outside the home	24	14
Lacking	23	13
**Origin of water**		
Public System	112	65
Well	53	30
River/lake	5	3
Other	3	2
**Sewage**		
Piped	25	14
Cesspit/Cesspool	131	76
Lacking	17	10
**Open ditch**		
Yes, near the homes	86	50
Yes, far from the homes	16	9
No	71	41

Most children 153 (88.4%) received both doses of the rotavirus vaccine, 12 (6.9%) received only the 1st dose, 3 (1.7%) received no dose and 5 (2.9%) showed no vaccination card.

1,113 stool samples were collected during the 12 months of follow-up, with 1,033 samples not associated and 80 associated with episodes of diarrhea. The average number of stool samples collected from each child over the 12 months was of 6.4 ± 3.5. There was a monthly average of 6.7 ± 3.0 episodes of diarrhea and an incidence of 0.5 ± 1.0 episode of acute diarrhea/child/year.

There was a higher incidence of infection by protozoa in the 1^st^ quarter of the study, with a subsequent decline as the children were getting older (4^th^ quarter). *Endolimax nana* was the most common protozoa in the 1^st^ quarter, and then, *Giardia lamblia*. Helminths, mostly *Ascaris lumbricoides* (5.5% and 6.8% in the 3^rd^ and 4^th^ quarters), were more frequent in the older children, while protozoa in the youngsters (Table 2). In diarrhea samples there was: four (5.0%) *A. lumbricoides*, three (3.75%) with the presence of *E. nana* and rotavirus and one (1.25%) with *T. trichiura* and *G. lamblia*. The remaining episodes of acute diarrhea are of unknown cause.

**Table 2:** Frequency of enteric infections by calendar quarter, Laranjeiras, Sergipe, Brazil, 2010-11

**Quarter**	**1st**		**2nd**		**3rd**		**4th**	**TOTAL**
**Number of cases**	n	%	n	%	n	%	n% n	%
*Trichuris trichiura*	2	0.9	0	0.0	0	0.0	0 0.0 2	0.2
*Ascaris lumbricoides*	11	5.2	7	2.7	18	5.5	21 6.8 57	5.1
*Entamoeba histolytica*	0	0.0	3	1.1	1	0.3	0 0.0 4	0.4
*Entamoeba coli*	0	0.0	2	0.8	2	0.6	4 1.3 8	0.7
*Endolimax nana*	27	12.7	4	1.5	3	0.9	0 0.0 34	3.0
*Giardia lamblia*	5	2.4	2	0.8	0	0.0	3 1.0 10	0.9
Rotavirus	1	0.5	4	1.5	17	5.2	7 2.3 29	2.6
TOTAL	46	21.7	22	8.4	41	12.5	35 11.4 144	13
**Total stool samples**	**212**		**263**		**330**		**308 1113**	

There was a lower frequency of rotavirus infection when the children were in the 1^st^ quarter (0.5%), with a progressive increase in incidence in the 3^rd^ (5.2%) and subsequent reduction in the 4th quarter of follow-up (2.3%). Rotavirus was identified in 29 (2.6%) of the 1,113 analyzed samples, with only three of them associated with a diarrhea episode (Table 2).

There was only one positive sample of pathogenic bacteria (*Shigella sp*) in the 666 stool samples analyzed (80 samples with diarrhea). During the 12-month follow-up, in the samples with diarrhea, rotavirus was present in 3.8% (3) cases. Enteroparasites were present in 11.3% of cases and *Ascaris lumbricoides* was the more frequently found. Logistic regression analysis could not identify influence of environmental conditions over the dependent variables episodes of diarrhea, rotavirus and parasitic infections (*p* > 0.05).

### Spatial analysis

**Figure 1:**
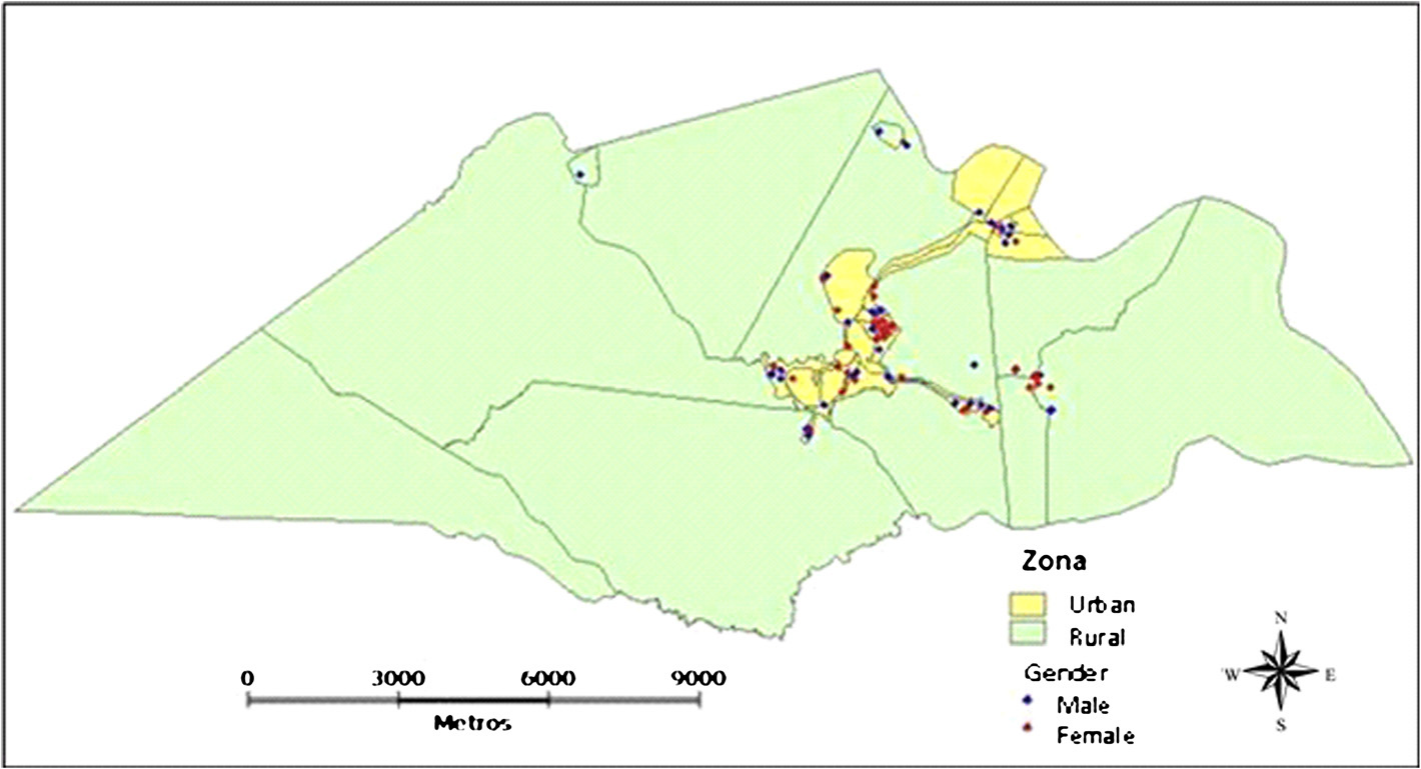
Map of the city of Laranjeiras with the distribution of children in study in rural and urban areas.

The interpolated surface shows a pattern of distribution of parasites, rotavirus and episodes of diarrhea ranging from mild to high. The Figure 1 shows the distribution of children in rural and urban areas.

**Figure 2:**
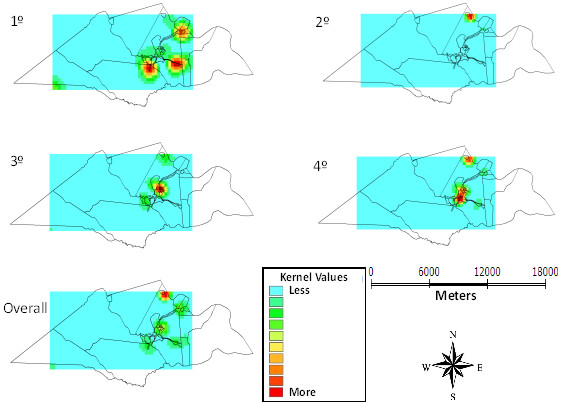
Kernel maps of the distribution of helminths per quarter and in 12 months, Laranjeiras, Sergipe, Brazil, 2010-11.

The frequency of helminths showed small variations between the quarters. In the 3rd and 4th quarters a highest number of cases occurred in the urban center and in the adjacent area, north of the region, represented by a rural village. Figure 2 shows the frequency of helminths in 12 months, where a similar distribution is observed among the study sites with case intensity between mild and moderate.

**Figure 3:**
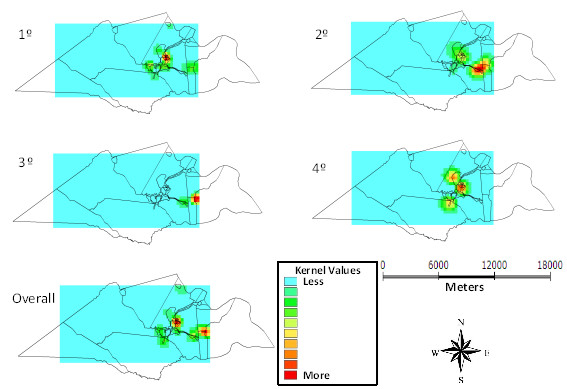
Kernel maps of the distribution of protozoa per quarter and overall in 12 months, Laranjeiras, Sergipe, Brazil, 2010-11.

Figure 3 shows that in the 1st quarter of the study, case intensity of protozoa infection was moderate to intense in the urban area, while in the other quarters the distribution ranged from mild and moderate risk across the whole region. The sum of 12 months showed that the frequency of protozoa is mild in most of the region, ranging from mild to moderate to the east, in the countryside, and in the urban center with the greatest case intensity.

**Figure 4:**
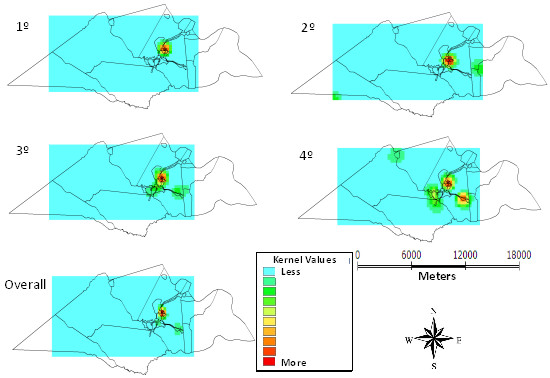
Kernel maps of the distribution of rotavirus per quarter and overall in 12 months, Laranjeiras, Sergipe, Brazil, 2010-11.

Rotavirus has appeared in all quarters predominantly in the urban center and ranging from mild to moderate in other areas. The frequency of rotavirus in the 12 months demonstrated case intensity from moderate to intense in the urban center and lower case intensity in the eastern region (Figure 4).

**Figure 5:**
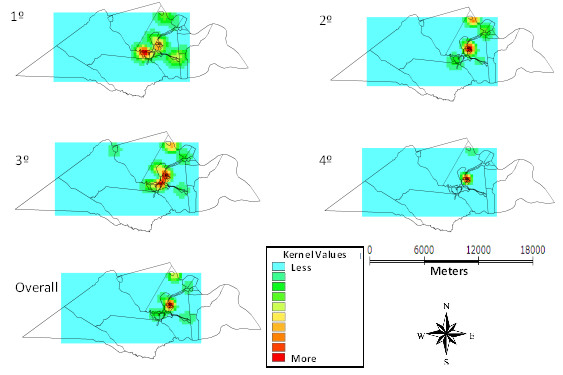
Kernel maps of the distribution of episodes of diarrhea per quarter and overall in 12 months, Laranjeiras, Sergipe, Brazil, 2010-11.

Figure 5 shows that the distribution of episodes of diarrhea is also more intense in the urban center and the areas relating to surrounding settlements presented frequency between mild and moderate.

## Discussion

This is the first study to describe the spatial distribution of acute diarrhea and enteric infections in a cohort of infants living in a low income community in Brazil with high population coverage of rotavirus vaccination. The number of acute diarrhea episodes/child/year was low and very few children had rotavirus diarrhea detected. These findings are similar to the ones found by Vieira et al. (2011) [20], that in an urban district of Aracaju city detected 0.87/episodes/child/year and 3.0% rotavirus associated diarrhea.

One of the particularities of this study was the occurrence of rotavirus in 2.5% of the stool samples without diarrhea, contrary to the findings of Borges et al. (2011) [21] that found no rotavirus among non-diarrhea stool samples. This finding suggests that the attenuating effect of the vaccine may be responsible for the subsequent asymptomatic infections, which is similar to original data from Mexico, where the natural rotavirus infection was partially protective against subsequent infection [22].

We observed a fluctuation on *A. lumbricoides* infection, but in the last period there was on increase (6,8%) in agreement with Roy et al. [23] in Bangladesh. The increasing prevalence of intestinal parasites by age observed in children is related to the process of child development (mobility and interaction with the environment) and longer exposure to environmental conditions [24]. During the 12 months of follow-up, 32 (25%) children may have been reinfected by parasites (protozoa or helminths), with 15 (47%) by *A.lumbricoides* more than once. During the 12 months of follow-up, 32 (25%) children may have been reinfected by parasites (protozoa or helminths), with 15 (47%) by *A.lumbricoides* more than once. We have not reassessed the children after treatment, but we have stressed to the families the importance for parasitic treatment and checked drug use.

Within protozoa, *E. nana* and *G.lamblia* were more prevalent and detected in earlier ages (4-13 months). *E. nana* is a commensal microorganism and it´s detection may indicate greater environmental contamination and other infestations may be found [25,26].

In our study, enteroparasites were also detected in diarrheic samples, similar to the findings from Nigeria, where 18.6% of toddlers with diarrhea were infected [27] and in Bangladesh, where 11.6% of infants had enteroparasites [23].

Most families in Laranjeiras, both in rural and urban areas, had water supplied by the public water system that used to work with constant discontinuity. Consequently, there was frequent storage of household water, most often improperly, compromising the quality of the water used by these families. The sanitary disposal ways, including the sewage, dumping of human waste and wastewater in public streams, were also poor. The homogeneity and frequency of these inadequate characteristics in the study area contributed to the significant occurrence of intestinal parasites, even in early childhood. Other studies have observed that such conditions are determinant to increase parasitic intestinal infections in different places [7,3,28].

The occurrences of parasites, rotavirus and diarrheal episodes in infants in this study have irregular distribution within the geographic space of Laranjeiras. The estimation of case intensity in the different areas by the Kernel method revealed higher frequency of enteric infections in the urban area and surrounding regions, where there is higher concentration of households and therefore a greater number of people living in an environment with poor sanitation.

One of the limitations of this study was the method of collection of stool samples, which depended on the availability of those responsible for the children in delivering monthly those samples, which may have contributed to an underestimation of the number of episodes of diarrhea. However, the children were visited at home at least every two weeks, which may have minimized the underestimation. Diarrhea episodes were not severe, and mothers may have not considered its occurrence as a problem and did not collect a sample.

## Conclusion

The results of this study demonstrate a low number of episodes of diarrhea in children, in a situation of poor environmental condition. This may suggest that high rotavirus vaccination coverage may have contributed for such a situation. It is also worthwhile to note the early occurrence of enteroparasitic infections, more frequent into the urban households, showing an evident environmental contamination that must be faced with better sanitary and living conditions for the population.

## Competing interests

The authors declare that they have no competing interests.

## Authors’ contributions

CBS: Contacting local health authorities and organizing field work; field work and data collection; analysis and interpretation of data; writing of manuscript; substantially revising it. KCA: Statistical analyses and interpretation of data; revising the paper. AJB: Contacting local health authorities and organizing field work; field work and data collection; analysis and interpretation of data; revising the paper. MBS: field work and data collection; analysis and interpretation of data. AR: Laboratory analyses and interpretation of data; revising the paper. SSD: Laboratory analyses and interpretation of data; revising the paper. RQG: Conception and design of the study; contacting local health authorities and organizing field work; interpretation of data; substantially revising it. All authors read and approved the final manuscript.

## Pre-publication history

The pre-publication history for this paper can be accessed here:

http://www.biomedcentral.com/1471-2458/14/399/prepub
